# Supplementary material for: Comprehensive Immune Monitoring of Clinical Trials to Advance Human Immunotherapy
Source: Cell Rep. 2019 Jul 16;28(3):819–831.e4. doi: 10.1016/j.celrep.2019.06.049 (PMC6656694; doi:10.1016/j.celrep.2019.06.049)
Supplement: Document S1. Figures S1–S4 and Tables S1–S3 [file mmc1.pdf]

Cell Reports, Volume *28*

Supplemental Information

Comprehensive Immune Monitoring of Clinical Trials to Advance Human Immunotherapy

Felix J. Hartmann, Joel Babdor, Pier Federico Gherardini, El-Ad D. Amir, Kyle Jones, Bita Sahaf, Diana M. Marquez, Peter Krutzik, Erika O'Donnell, Natalia Sigal, Holden T. Maecker, Everett Meyer, Matthew H. Spitzer, and Sean C. Bendall

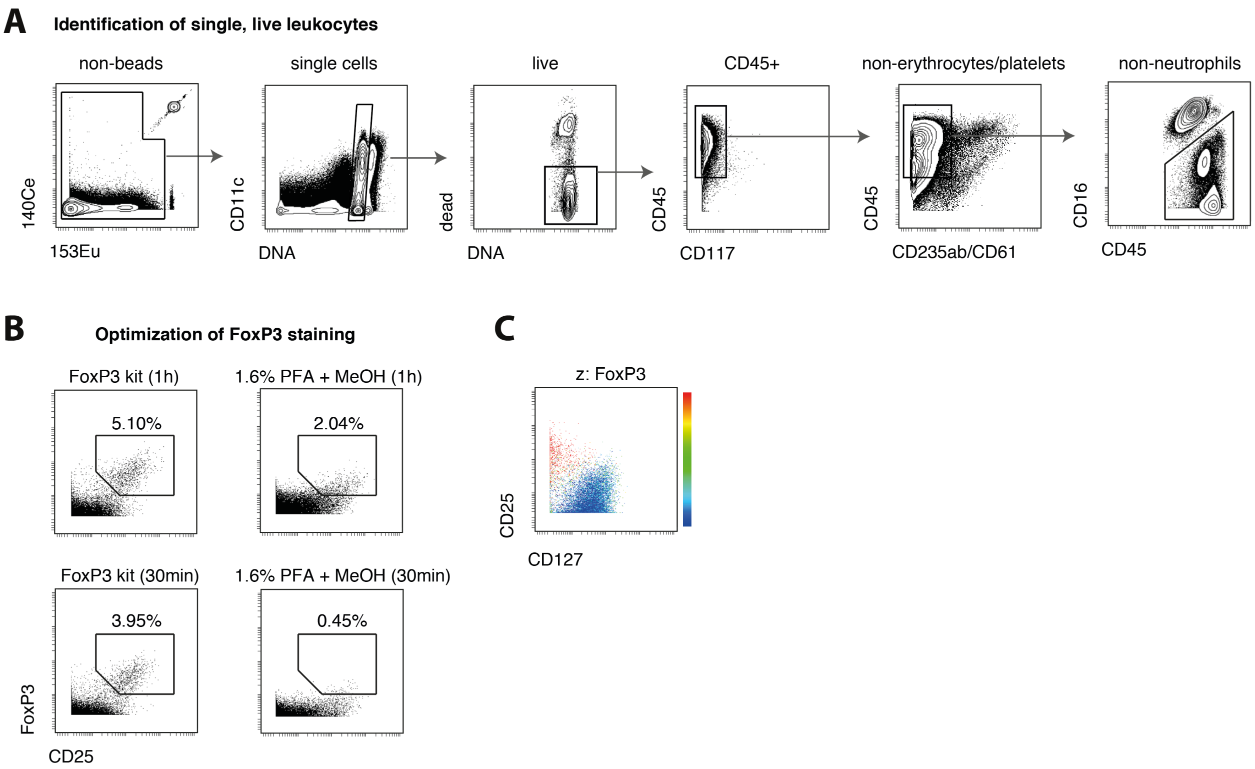

Figure S1. Identification of single, live leukocytes and optimization of FoxP3 staining. **Related to Figure 2.** PBMCs or whole blood of healthy donors was stained with the reference panel as outlined in Table S1. (A) Serial gates used to identify single, live cells. Firstly, remaining beads are excluded and next, DNA positive events are identified. CD11c is used to discriminate duplets from monocyte populations which show higher DNA staining. Next, cisplatin-positive (dead) cells are excluded and CD45+ (non-mast cells) are selected. Potentially remaining erythrocytes, platelets and neutrophils are excluded. Representative examples shown here may be from different samples to demonstrate the utility of each gating step. (B) PBMCs from healthy subjects were first stained for surface antigen and then fixed using different fixation schemes. For the FoxP3 fixation permeabilization kit (left), cells were fixed with 1x fixation buffer for 1 h at RT. Intracellular staining was performed for 1 h (top) or 30 min (bottom) in 1x permeabilization buffer. Alternatively, cells were fixed with 1.6% PFA (right) for 10 min at RT and permeabilized with 100% cold MeOH for 10 min on ice. Cells were then stained intracellularly for 1 h (top) or 30 min (bottom) in CSM. Shown are $CD4^+$ T cells. (C) Cells are colored by FoxP3 intensity as well as CD127 and CD25 which can be used to identify Treg cells.

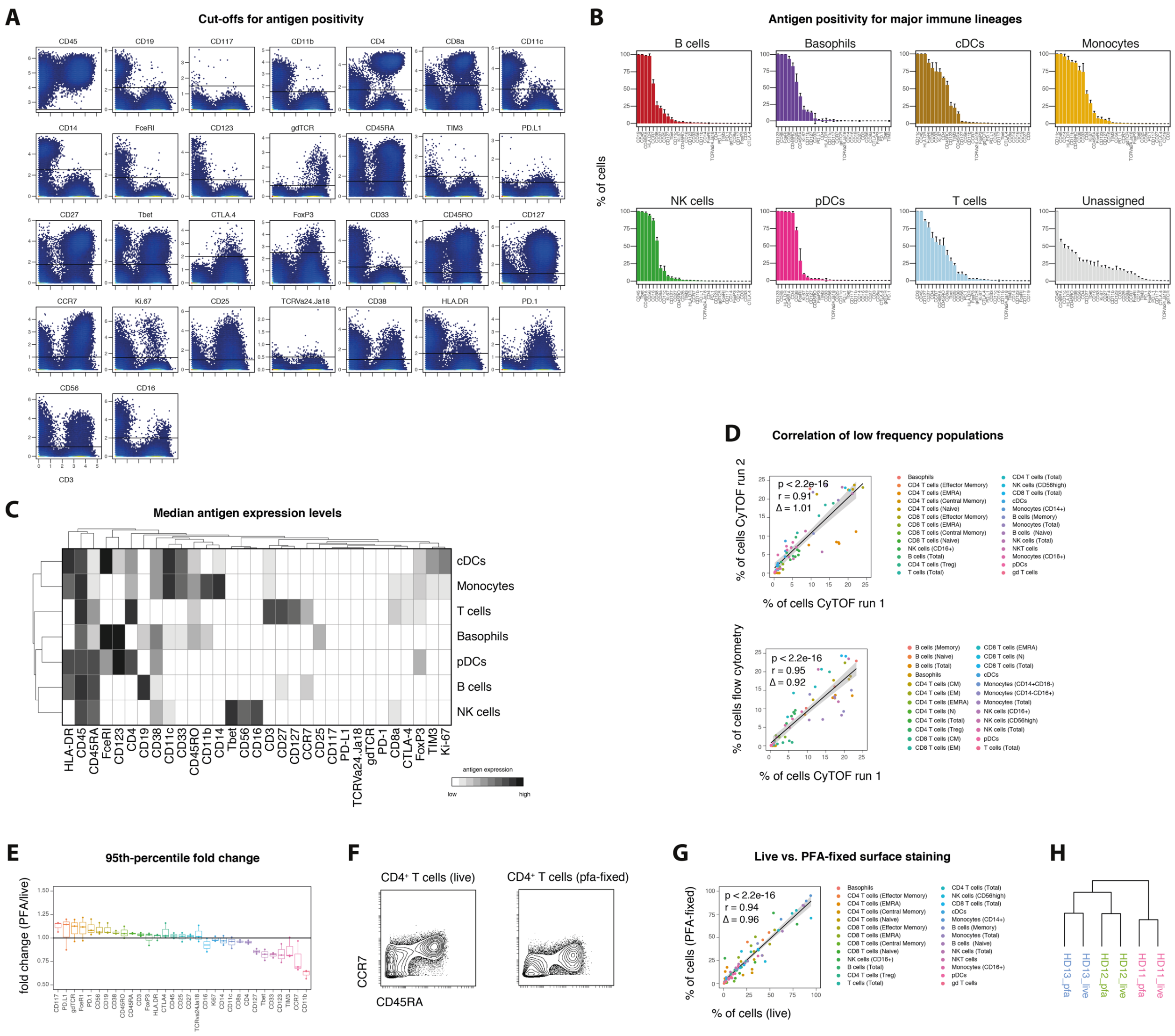

Figure S2. Robustness of immune cell lineage identification. Related to Figure 3. PBMCs from healthy donors (n = 5) were stained with the reference panel and analyzed by mass cytometry. Cells were pre-gated as single, live, CD45$^+$, non-neutrophils. (A) Cutoffs (black horizontal lines) used to determine positivity of antigen expression. (B) Median percentage of cells in each immune cell lineage which is positive for the indicated antigen based on the cutoffs as in A. Error bars represent s.e.m. (C) Median antigen expression levels on manually gated immune cell lineages. (D) Immune cell frequencies (only populations below 25% of total cells are shown) determined by two different CyTOF analyses (top) or by CyTOF and flow cytometry (bottom). Frequencies of immune lineages were determined through serial gating. Linear regression line is shown in black with the 95% confidence intervals (CI, shaded). Coefficients, p-values and slope Δ were calculated based on data from all donors. (E-H) PBMCs from healthy donors (n = 3) were stained directly with surface antibodies or fixed with 1.6% PFA for 10 min at RT before

surface staining. (E) To assess the influence of PFA-fixation on the maximum dynamic range, we first calculated the 95th percentile of each marker and subsequently calculated the respective fold change (FC) for all markers. The black horizontal line represents FC = 1. Boxplots depict the interquartile range (IQR) with a horizontal line representing the median. Whiskers extend to the farthest data point within a maximum of 1.5x IQR. Points represent individual samples. (F) Exemplary biaxial plots showing reduced CCR7 and comparable CD45RA staining intensities on fixed T cell populations. (G) Frequencies of immune cell populations were determined through serial gating and plotted against each other. Linear regression line is shown in black with the 95% confidence intervals (CI) shaded. Coefficients, p-values and slope Δ were calculated based on data from all donors. (H) Hierarchical clustering of unfixed or fixed samples based on frequencies as in G.

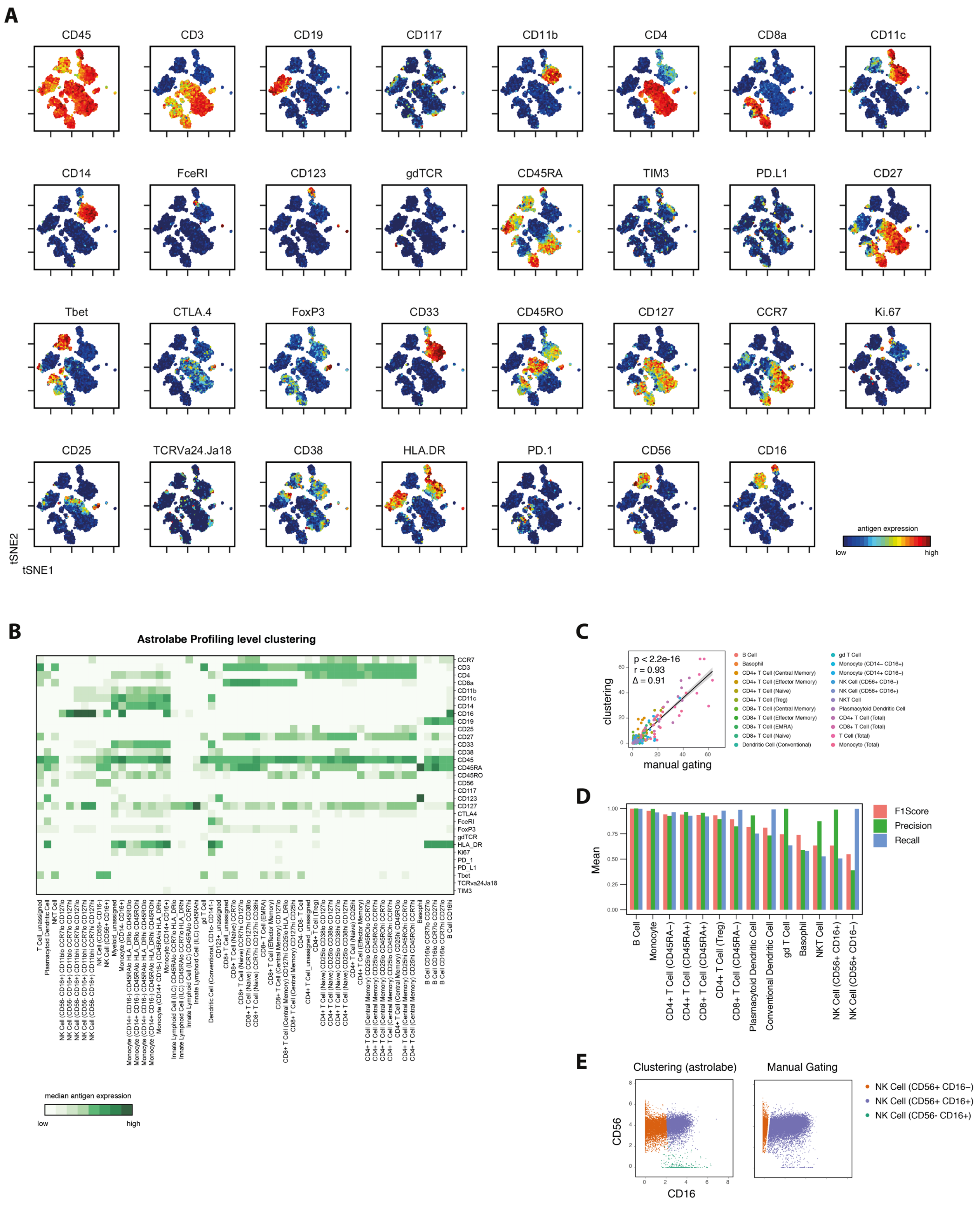

Figure S3. Automated profiling of immune cell populations. Related to Figure 4. (A) tSNE visualizations were produced from randomly subsampled data (20'000 cells) of five healthy donors. Indicated antigen expression levels are overlaid as a color dimension. (B) PBMC data from healthy donors was clustered and automatically annotated

using the Astrolabe platform. Shown are median expression levels of all antigens across all clusters on the Profiling level. (C) Frequencies of immune cell populations determined by manual gating or through Astrolabe clustering. Linear regression line is shown in black with the 95% confidence intervals (CI) shaded. Coefficients, p-values and slope Δ were calculated based on data from all donors. (D) Mean precision, recall and F1score between manual lineage assignments and Astrolabe-based clustering across different populations. (E) Example of differential thresholds employed in clustering (left) and manual gating (right).

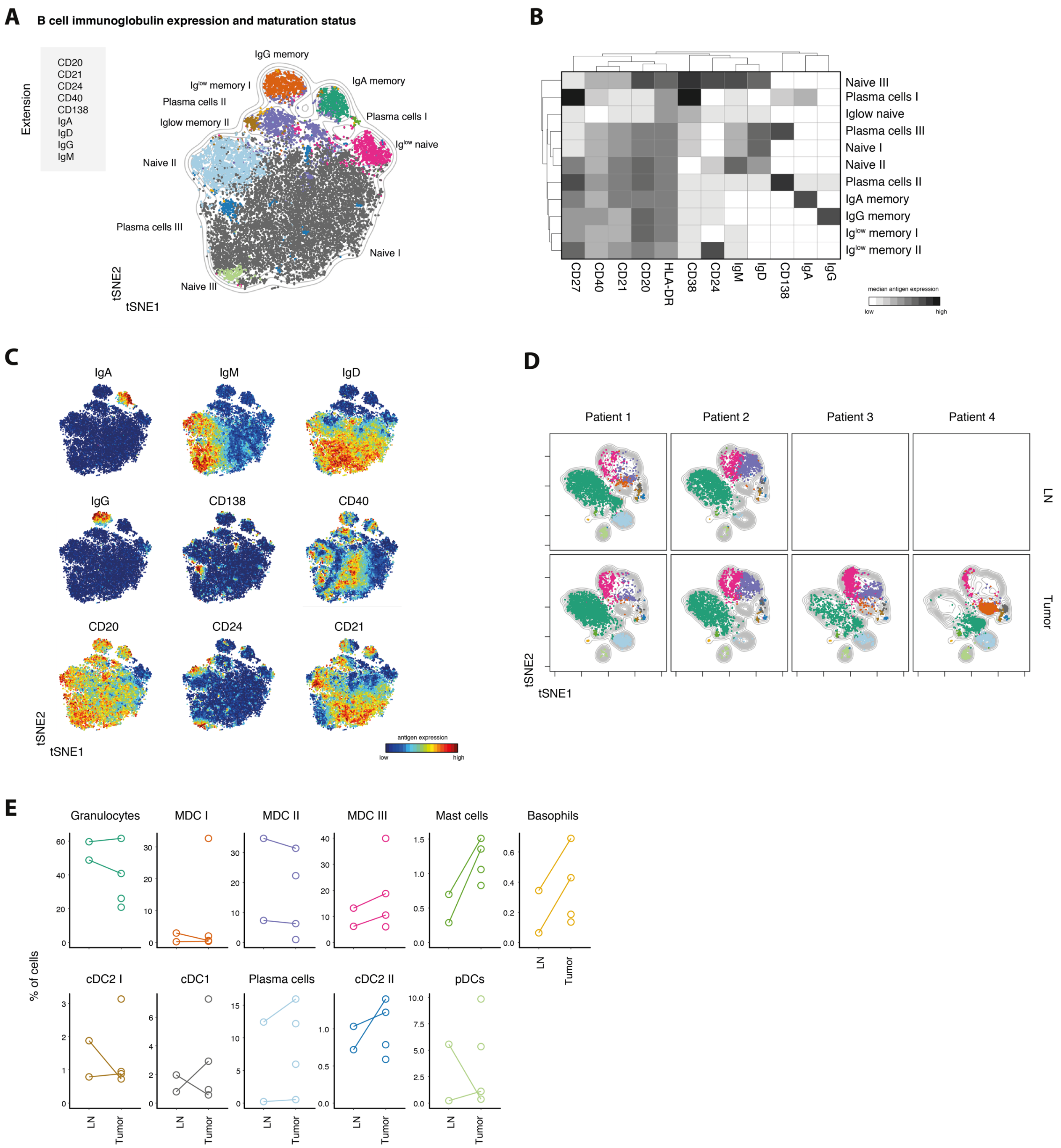

Figure S4. Exploration of heterogeneous populations through panel extensions. Related to Figure 6. (A) Antibodies targeting additional antigens of interest (left) were conjugated to non-occupied heavy metal isotopes. PBMCs from healthy donors (see Table S2) were stained with these antibodies in combination with the reference set. Data was pre-gated to exclude T cells and NK cells. To create a tSNE overview, data from all samples was randomly subsampled to 21’000 cells with equal contribution from all samples. Cells are colored by their FlowSOM-based cluster-assignment. Grey lines indicate the density distribution of the tSNE map. (B) Cluster-based

median expression levels for all population relevant antigens used in the tSNE and FlowSOM analysis. (C) Protein expression levels of all additional antigens are overlaid as a color-dimension onto the tSNE map. (D-E) Antibodies targeting additional myeloid antigens were conjugated to non-occupied heavy metal isotopes. Cells from lymph node biopsies (n = 2) and tumor biopsies (n = 4) of patients with head and neck carcinoma (see Table S2) were stained with these antibodies in combination with the reference set. (D) tSNE overview with colors denoting FlowSOM-cluster assignments stratified by sample. (E) Frequencies of FlowSOM-based clusters as in D in all samples. Lines connect different tissues of the same patients.

Table S1. Reference panel of anti-human antibodies for mass cytometry. Related to Figure1.

Isotope	Element	Marker	Clone	Staining step	Dilution factor	Catalogue number
89	Y	CD45	H130	surface	100	3089003B
102	Pd					
104	Pd					
105	Pd					
106	Pd					
108	Pd					
110	Pd					
113	In					
115	In					
139	La	CD235ab/CD61	HIR2/VI-PL2	surface		custom
140	Ce					
141	Pr	CD3	UCHT1	surface		custom
142	Nd	CD19	HIB19	surface	100	3142001B
143	Nd	CD117	104D2	surface	100	3143001B
144	Nd	CD11b	IRCF44	surface	400	3144001B
145	Nd	CD4	RPA-T4	surface	200	3145001B
146	Nd	CD8a	RPA-T8	surface	1600	3146001B
147	Sm	CD11c	BU15	surface	200	3147008B
148	Nd	CD14	RMO52	surface	1600	3148010B
149	Sm					
150	Nd	FceRI	AER-37 (CRA-1)	surface	100	3150027B
151	Eu	CD123	6H6	surface	100	3151001B
152	Sm	gdTCR	11F2	surface	100	3152008B
153	Eu	CD45RA	HI100	surface	100	3153001B
154	Sm	TIM3	F38-2E2	surface	100	3154010B
155	Gd					
156	Gd	PD-L1 (CD274)	29E.2A3	surface	100	3156026B
157	Gd					
158	Gd	CD27	L128	surface	400	3158010B
159	Tb					
160	Gd	Tbet	4B10	intracellular	100	3160010B
161	Dy	CD152 (CTLA-4)	14D3	intracellular	800	3161004B
162	Dy	FoxP3	PCH101	intracellular	400	3162011A
163	Dy	CD33	WM53	surface	100	3163023B
164	Dy	CD45RO	UCHL1	surface	200	3164007B
165	Ho	CD127	A019D5	surface	100	3165008B
166	Er					
167	Er	CCR7 (CD197)	G043H7	surface	100	3167009A
168	Er	Ki-67	B56	intracellular	400	3168007B
169	Tm	CD25	2A3	surface	100	3169003B
170	Er	TCR Va24-Ja18	6B11	intracellular	100	3170015B
171	Yb					
172	Yb	CD38	HIT2	surface	400	3172007B
173	Yb					
174	Yb	HLA-DR	L243	surface	100	3174001B
175	Lu	PD-1	EH12.2H7	surface	100	3175008B
176	Yb	CD56	NCAM16.2	surface	100	3176008B
191	Ir	DNA				
191	Ir	DNA				
194	Pt					
195	Pt	Live/dead				
196	Pt					
198	Pt					
209	Bi	CD16	3G8	surface	100	3209002B

Table S2. Donor characteristics. Related to Figure 3, Figure 4, Figure 5 and Figure 6.

Healthy donors

Donor_ID	Tissues_analyzed	Diagnosis	Age	Sex
HD01	PBMC	Healthy Donor	NA	NA
HD02	PBMC	Healthy Donor	NA	NA
HD03	PBMC	Healthy Donor	NA	NA
HD04	PBMC	Healthy Donor	NA	NA
HD05	PBMC	Healthy Donor	NA	NA
HD06	PBMC	Healthy Donor	39	M
HD07	PBMC	Healthy Donor	35	M
HD08	PBMC	Healthy Donor	46	F
HD09	PBMC	Healthy Donor	31	F
HD10	PBMC	Healthy Donor	54	F
HD11	PBMC	Healthy Donor	NA	NA
HD12	PBMC	Healthy Donor	NA	NA
HD13	PBMC	Healthy Donor	NA	NA

Tumor

Donor_ID	Tissues_analyzed	Diagnosis	Age	Sex	Tumor_site	Tumor	p16_tatus	Locoregional LN Met	AJCC Stage
T01	Tumor	SCC	57	M	Tonsil	Primary	Positive	Yes	pT2N2b
T02	Tumor, Met	SCC (poor)	78	F	Tongue	Recurrent	NA	Yes	pT3N2c
T03	Tumor, Met	SCC (moderate)	83	F	Gingiva	Primary	NA	Yes	pT4aN2b
T04	Tumor	SCC (moderate)	69	F	Floor of mouth	Primary	NA	Yes	pT4aN2c
T05	Tumor	SCC (basaloid)	55	M	Supraglottis	Recurrent	NA; prior was positive	NA	pT4aNx
T06	Tumor	SCC (moderate to poor)	70	M	Ventral tongue / Floor of	Primary	NA	Yes	pT2N1
T07	Tumor	Warthin Tumor	76	M	Parotid Gland	Primary	NA	No	NA

BMT patients

Donor_ID	Tissues_analyzed	Diagnosis	Age	Sex	aGVHD	GVHD_onset	cGVHD	SAE	CMV (D/R)	Conditioning	GVHD PPx	Graft_type	Sampling
BMT01	PBMC	AML	58	F	None	NA	None	E coli. bacteremia	+/+	Bu/Cy	Tacrolimus, MTX	PBMSC	day_30, day_90
BMT02	PBMC	AML	57	M	None	NA	None	Coccoides infection, CMV	+/+	Bu/Cy	Tacrolimus, MTX	PBMSC	day_30, day_90
BMT03	PBMC	B cell ALL	31	F	None	NA	None	Tacrolimus associated	-/-	TBI/VP-16	Tacrolimus, MTX	PBMSC	day_30, day_90
BMT04	PBMC	AML	39	F	None	NA	None	Clostridrium bacteremia,	+/+	TBI/VP-16	Tacrolimus, MTX	PBMSC	day_30, day_90
BMT05	PBMC	AML	58	F	None	NA	None	None	+/+	Bu/Cy	Tacrolimus, MTX	PBMSC	day_30, day_90
BMT06	PBMC	FLT3+ AML	42	M	None	NA	None	Mild VOD	+/-	Bu/Cy	Sirolimus	CD34/Treg/Tcon	day_30, day_90
BMT07	PBMC	ETP-ALL	34	M	None	NA	None	Herpes labialis	+/+	TBI/Cy/VP-16	Sirolimus	CD34/Treg/Tcon	day_30, day_90
BMT08	PBMC	ALL	20	M	None	NA	None	Acute cholecystitis,	+/+	Bu/Cy	Tacrolimus	CD34/Treg/Tcon	day_30
BMT09	PBMC	CML blast crisis	54	M	None	NA	None	Coronavirus URI	+/-	Bu/Cy	Tacrolimus	CD34/Treg/Tcon	day_30, day_90
BMT10	PBMC	AML	53	M	None	NA	None	Unspecified transaminitis	-/-	Bu/Cy	Tacrolimus	CD34/Treg/Tcon	day_30
BMT11	PBMC	MF	56	M	None	NA	None	Strep mitis bacteremia	+/-	Bu/Cy	Tacrolimus	CD34/Treg/Tcon	day_30, day_90
BMT12	PBMC	Refractory NHL	49	M	None	NA	None	CoNS bacteremia	-/-	BCNU/VP-16/Cy	Sirolimus	CD34/Treg/Tcon	day_30, day_90
BMT13	PBMC	AML	61	F	Yes, grade 3 skin and liver	day_22	Mild day_474	CMV reactivation	-/-	TBI/Cy/VP-16	None	CD34/Treg(frozen)/T	day_30, day_90
BMT14	PBMC	Gamma delta NHL	45	F	Yes, grade 1 skin	day_30	Mild day_145	Gram negative bacteremia	+/+	BCNU/VP-16/Cy	None	CD34/Treg(frozen)/T	day_30, day_90
BMT15	PBMC	AML	51	M	Yes, grade 1 skin	day_24	None	Acute diverticulitis, HHV6	+/+	TBI/Cy/VP-16	None	CD34/Treg(frozen)/T	day_30, day_90

Table S3. Extension panels. Related to Figure 6.

B cell immunoglobulin expression and maturation status

Isotope	Element	Marker	Clone	Staining step
113	In	CD40	5C3	surface
115	In	CD20	2H7	surface
140	Ce	IgM	G20-127	surface
149	Sm	IgA	IS11-8E10	surface
155	Gd	IgD	IA6-2	surface
157	Gd	CD138	DL-101	surface
159	Tb	IgG	M1310G05	surface
166	Er	CD24	ML5	surface
171	Yb	CD21	LT21	surface
173	Yb	CD40L	24-31	surface

Myeloid cell diversity across multiple tissues

Isotope	Element	Marker	Clone	Staining step
113	In	CD66cd	YTH71.3	surface
115	In	CD7	M-T701	surface
140	Ce	CD86	IT2.2	surface
149	Sm	CD1c	L161	surface
155	Gd	CD64	10.1	surface
157	Gd	CD206	15-2	surface
159	Tb	2B4	M2B4	surface
164	Dy	CD172ab	SE5A5	surface
166	Er	CD141	1A4	surface
171	Yb	CD40	5C3	surface
173	Yb	CCR2	K036C2	surface
